# New Approach to Optimize Mechanical Properties of the Immiscible Polypropylene/Poly (Ethylene Terephthalate) Blend: Effect of Shish-Kebab and Core-Shell Structure

**DOI:** 10.3390/polym10101094

**Published:** 2018-10-02

**Authors:** Yingxiong Wang, Dashan Mi, Laurens Delva, Ludwig Cardon, Jie Zhang, Kim Ragaert

**Affiliations:** 1 College of Polymer Science and Engineering, State Key Laboratory of Polymer Materials Engineering Sichuan University, Chengdu 610065, China; wang137236180@163.com (Y.W.); Dashan.Mi@ugent.be (D.M.); 2 Centre for Polymer and Material Technologies, Department of Materials, Textiles and Chemical Engineering, Faculty of Engineering and Architecture, Ghent University, Technologiepark 915, 9052 Ghent, Belgium; Laurens.Delva@ugent.be (L.D.); Ludwig.Cardon@ugent.be (L.C.)

**Keywords:** Shish-kebab, core-shell structure, PP/PET blend, solid plastic waste

## Abstract

Improving the mechanical properties of immiscible PP/PET blend is of practical significance especially in the recycling process of multi-layered plastic solid waste. In this work, a multi-flow vibration injection molding technology (MFVIM) was hired to convert the crystalline morphology of the PP matrix from spherulite into shish-kebab. POE–*g*–MA was added as compatibilizer, and results showed that the compatibilization effect consisted in the formation of a core-shell structure by dispersing the POE–*g*–MA into the PP matrix to encapsulate the PET. It was found that the joint action of shish-kebab crystals and spherical core-shell structure enabled excellent mechanical performance with a balance of strength and toughness for samples containing 10 wt % PET and 4 wt % POE–*g*–MA, of which the yield strength and impact strengths were 50.87 MPa and 13.71 kJ/m^2^, respectively. This work demonstrates a new approach to optimize mechanical properties of immiscible PP/PET blends, which is very meaningful for the effective recycling of challenging plastic wastes.

## 1. Introduction

The lifecycle of polymer materials leads to two major types of solid plastic wastes (SPW), namely post-industrial (PI) waste and post-consumer (PC) waste [1], which are generated at the processing stage and the end-of-life, respectively. From the environmental point of view, in comparison with landfill or energy recover, the preferable way to handle these wastes is recycling, which is often divided into mechanical and chemical recycling. The mechanical recycling, including collection, sorting, washing, and grinding of materials, has been developed as the most common method for recycling of SPW [2,3]. 

The recycling of poly (ethylene terephthalate) (PET) or polypropylene (PP) as separate mono-materials (after sorting) is a typical example of a broadly utilized mechanical recycling process, especially for packaging waste [3]. However, in some cases, it is not possible to simply sort out the single materials from other contaminating plastic waste. Examples of this include industrial carpet waste and multilayered packaging waste. The former contains PET yarns physically attached to a PP backing [4], while in the latter PET provides barrier properties and laminated PP provides water resistance as well as sealing ability. Thus, melt blending is the only viable pathway for mechanical recycling.

Due to both economic and academic significance, blending PP with PET has drawn much attention [5,6,7,8,9,10]. However, the thermodynamic immiscibility of PP/PET blend is a major obstacle, which leads to a two-phase morphology with poor interfacial adhesion. The key issues for the production of high-performance immiscible polymer blend cover the following aspects: (i) Homogeneous dispersion and embedding of dispersed phase in matrix; (ii) strong interfacial interaction between the dispersed phase and matrix; and (iii) adequate morphology design for dispersed phase and matrix. Here, in case of the PP/PET blend, the morphology refers to both the phase morphology of the PET and the crystalline morphology of the PP. 

The dispersed phase can possess different morphologies [11], such as spherical drop, cylinder, fiber, sheet, or co-continuous phase. The resulting morphology is partly determined by processing conditions (external fields such as temperature and pressure). In contrast to PET fibers induced by special stretching technology, conventional processing methods only result in the formation of spherical or slightly elongated droplets. From the thermodynamic point of view, the average size of the dispersed phase is determined by the interfacial tension [12], which can be mitigated by the introduction of compatibilizers. Reduced domain size of the dispersed phase is beneficial for the mechanical properties of a PP/PET blend, especially the impact strength. The efficiencies of various compatibilizers have been widely discussed [4,8,13] and the criteria for selecting compatibilizers for PP/PET blend can be briefly summarized as: (i) The material should show affinity to both components of the immiscible blend; and (ii) as a third component, the modification effect of compatibilizer itself on mechanical properties of PP/PET should also be considered. Following these rules, elastomer-based compatibilizers, such as polyolefin grafted maleic anhydride (POE–*g*–MA) or styrene ethylene butylene styrene grafted maleic anhydride (SEBS–*g*–MA) are preferable since additional impact modification effect is expected due to the elastomeric character of the POE and SEBS backbone. However, loading soft elastomers would also lead to a deterioration of modulus and tensile strength, which is not beneficial for the balance between strength and toughness. Fortunately, these elastomer-based compatibilizers will preferentially locate themselves at the interface of PP and PET due to the interaction between anhydride and carbonyl groups [14] and the compatibility of their backbone and the PP matrix, resulting in the formation of a core-shell structure with elastomeric shell and PET core. To the best of our knowledge, there is rare report focusing on the mechanical properties of PP/PET blend in the view of the core-shell structure. Kordjazi [13] observed the core-shell structure in PP/PET/SEBS–*g*–MA blend, but he concentrated on the rheology behavior instead of mechanical property. In general, the core-shell structure is supposed to maintain the balance between toughness and strength since only a small load of elastomers can achieve high toughness with the aid of a core-shell structure [15]. It is needed to figure out the influence of the morphology for the core-shell structure on mechanical properties of PP/PET blends with elastomer-based compatibilizers. 

The crystalline morphology of the PP also plays an important role in determining the final properties of the PP/PET blend. Under different processing conditions, PP can form various crystalline morphologies, such as spherulite, shish-kebab, and cylindrite, among which shish-kebab has been profoundly investigated covering its formation mechanism [16,17,18] and the effect on improving mechanical properties [19,20,21]. It is found that a high content of shish-kebab will significantly improve tensile strength and impact strength of isotactic PP (iPP) simultaneously [20]. However, due to some technical limits, it lacks adequate research to introduce sufficient shish-kebab to reinforce a PP/PET blend. Zhong [22] has incorporated shear controlled orientation injection molding (SCORIM), which was developed by Bevis et al. [23,24], to study PP with PET microfibril network. Results showed that tensile strength of the blend increased from 37 MPa of conventional injection molded samples to 49 MPa of SCORIM samples, which was lower than that of neat PP with an increase from 42 to 53 MPa. SCORIM could promote the formation of shish-kebab and thus increase tensile strength. However, the incorporation of PET microfibers deteriorated it, as compared with neat PP due to the incompatibility of PP and PET.

For the unavoidably blended PP/PET solid polymer waste with a minor content of PET, compared with PET microfibers, it is better to take advantage of the existing PET to form a core-shell structure by loading a small content of elastomeric compatibilizer, towards a better compatibility and higher impact toughness. However, the strong shear force needed for the formation of PP shish-kebab crystals may also result in PET fibers or elongated PET droplets, which hinders the formation of the core-shell structure. In this experiment, to obtain PP/PET blends containing core-shell structure and shish-kebab simultaneously, POE–*g*–MA was added and the facile preparation of shish-kebab with core-shell structure was achieved by utilizing a self-developed multi-flow vibration injection molding technology (MFVIM) [25,26,27], which can provide a periodical strong shear field to the polymer melt in the mold cavity at packing stage. Especially, melt temperature for injection molding was set as 200 °C, at which PET was not fully molten, and thus was supposed to not easily be deformed during the process. As far as we know, this is the first report to take advantage of both shish-kebab and core-shell structure to reinforce a PP/PET blend with a balance between strength and toughness. The morphology evolution of the core-shell structure and its influence on final properties were studied by changing the content of PET and POE–*g*–MA. It was found that shish-kebab and core-shell structure could jointly improve mechanical properties and maintain strength-toughness balance. This work demonstrates a new approach to improve mechanical performance of a PP/PET blend with a potential application in the upcycling of recycled PSW.

## 2. Experimental 

### 2.1. Materials

Polypropylene (PP) was purchased from Sabic (Sabic 575P, Bergen op Zoom, The Netherlands) with a melt flow rate (MFR) of 11 g/10 min (2.16 kg, 230 °C). PET (trade name LIGHTER C93), which is a bottle-grade material with an intrinsic viscosity of 0.80 ± 0.02 dL/g, was provided by Equipolymers (Schkopau, Germany). POE–*g*–MA used in this study was Acti-Tech 16MA13, which is a Vistamaxx-based compatibilizer with a density of 0.882 g/cm^3^, kindly donated by Nordic Grafting Company (NGC, Hellerup, Denmark). The grafting percentage of the MA group onto the backbone of the compatibilizer was 1.3 wt %, according to the data sheet. PET was dried in a vacuum oven for 15 h at 80 °C, and 2 h before processing at 120 °C.

### 2.2. Sample Preparation

Melt blending was achieved by a twin-screw extruder (Coperion ZSK18, Stuttgart, Germany) with two co-rotating screws of 18 mm diameter, *L*/*D* = 40 and a die opening of 19 mm × 2 mm. The screw speed was set at 120 rpm and the barrel temperatures were set between 205 and 260 °C. The mixture of solid materials was directly added into extruder after simple mechanical mixing. The extrudate was obtained as a sheet with dimensions of 25 mm × 1 mm, by passing through calender rolls, which were cooled down to 15 °C. Then, the sheet was shredded prior to injection molding. The composition of the different blends is listed in Table 1 and the blends are named as *x* or *x*-C, in which *x* refers to the weight percent of PET in the PP/PET binary blend and -C means that the blend contains compatibilizer. It should be noted that the weight percent of POE–*g*–MA was calculated in the PP/PET/POE–*g*–MA ternary blend. 

The compounded materials were then transferred into injection molding on a home-made injection machine after dried in vacuum oven for 2h at 100 °C. In this experiment, multi-flow vibration injection molding (MFVIM) and conventional injection molding (CIM) were hired to fabricate samples with different content of shish-kebab, which were named as VIM-*x*(-C) and CIM-*x*(-C), respectively. Both MFVIM and CIM were carried out on the same machine. The only difference between MFVIM and CIM is that during packing stage an extra vibration pressure with an amplitude of 60 MPa and a frequency of 0.55 Hz was provided for MFVIM while for CIM the packing pressure kept constant at 30 MPa. The detailed information about the difference of MFVIM and CIM has been introduced in previous work [20,26]. Temperature profile (°C) from hopper to nozzle was 160, 180, 190, 200, and 200 and mold temperature was set as 40 °C. Dumbbell bars (50 × 9 × 3 mm^3^, with a gauge length of 20 mm) for tensile test and strip bars (60 × 10 × 3 mm^3^) for impact test were cut from molded square sheet (60 × 60 × 3 mm^3^) along the flow direction.

It should be noted that the content of compatibilizer used in this experiment varied for different samples, namely 2%, 4% and 6% for CIM/VIM-5-C, CIM/VIM-10-C and CIM/VIM-20-C respectively. As an elastomer the content of POE–*g*–MA also influence the final properties, so it is necessary to explain why this content was chosen. Figure S1 (Supplementary Materials) proves that only 2% POE–*g*–MA is enough for 5% PET, and at least 6% POE–*g*–MA is needed for 20% PET to achieve sufficient compatibilization. The details can be found in Supplementary Material.

### 2.3. Polarized Light Microscopy (PLM)

**Figure 1:**
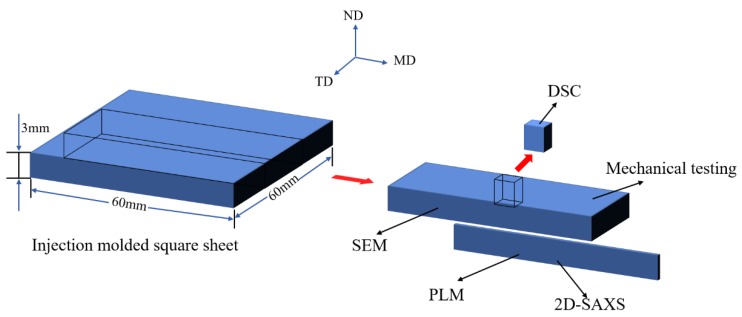
The schematic drawing of sampling methods for each analyzes (MD: Flow direction, TD: Transverse direction, and ND: Normal direction.).

Thin slices with thickness of 30 μm were cut along flow direction by microtome. Then the slices were observed by DX-1 (Jiang Xi Phoenix Optical Co. China) microscope connected with a Nikon 500D digital camera. Detailed sampling method is shown in Figure 1.

### 2.4. Two-Dimensional Small Angle X-ray Scattering (2D-SAXS)

2D-SAXS was applied to detect the changes in morphology of PP matrix, using a scatterometer (Xeuss2.0, Xenocs, Sassenage, France). The specimens were 1 mm thick slices cut from molded square sheets along the flow direction. The X-ray beam, with a wavelength of 0.154 nm, was created by a Cu tube. The sample-to-detector distance for SAXS was 2474 mm, and the exposure time was set as 300 s for each sample. 

### 2.5. Scanning Electron Microscopy (SEM)

A JEOL field emission scanning microscopy (model JSM-7500F, Tokyo, Japan) with an acceleration voltage of 20 kV was employed for detailed morphology observation. Specimens were cut parallel to the flow direction. To carefully observe the crystal morphology of PP and phase morphology of dispersed PET, the specimens were etched by acid solution of H_2_SO_4_–H_3_PO_4_–KMNO_4_ at 50 °C for 12 h. Besides, specimens including POE–*g*–MA were etched in n-heptane at 50 °C for 7 h to remove POE–*g*–MA to observe the core-shell structure. All the specimens were dried and then coated with a thin layer of gold prior to SEM characterization. 

### 2.6. Differential Scanning Calorimetry (DSC)

The melting behavior of the PP matrix for the different samples was analyzed by a DSC device (TA Q200) in the temperature range between 40 to 200 °C with a heating rate of 10 °C/min. The following equation was utilized for calculating the total crystallinity *X*_c_ for PP of each sample:

(1) $$\;Xc=\frac{\Delta H_{m}}{\Delta H_{m}^{0}\varphi_{i}}\;$$

in which $\Delta H_{m}$ represents the measured fusion enthalpy and $\Delta H_{m}^{0}$ is the fusion enthalpy of completely crystallized PP. Here the value of $\Delta H_{m}^{0}$ is selected as 207 J/g. $\varphi_{i}$ is the mass fraction of PP in the blend. The samples were cooled to 40 °C with a cooling rate of 10 °C/min for non-isothermal crystallization after isothermal at 200 °C for 10 min. The peak temperature during non-isothermal crystallization was recorded as crystallization temperature *T*_c_. All the DSC measurements were carried out under dry nitrogen atmosphere.

### 2.7. Dynamic Rheology Measurement

Materials were firstly compressed into disks (diameter of 20 mm and thickness of 1 mm) at 200 °C with the pressure of 10 MPa before rheology tests. Dynamic rheology measurements were carried out on a parallel-plate rotational rheometer (Thermo Scientific MARS III, Haake, Karlsruhe, Germany). The diameter of the plate is 20 mm, and the gap between the two plates is 0.8 mm. For all samples, measurements were performed at 200 °C covering a frequency range from 0.01 Hz to 100 Hz with a strain of 1%, after an isothermal of 5 min between the gap of the two plates to erase the thermal-mechanical history.

### 2.8. Mechanical Testing

Tensile properties (yield strength and Young’s modulus) along flow direction were measured by an Instron 5967 machine with a cross-head speed of 20 mm/min. Notched Izod impact strength was used to evaluate the toughness of the samples. The impact tests were performed on a XJUD-5.5 Izod machine and a 2 mm deep V-shaped notch was made for each specimen before test. All the mechanical properties were measured and calculated as the average over six samples. 

## 3. Results and Discussion

### 3.1. Mechanical Properties

**Figure 2:**
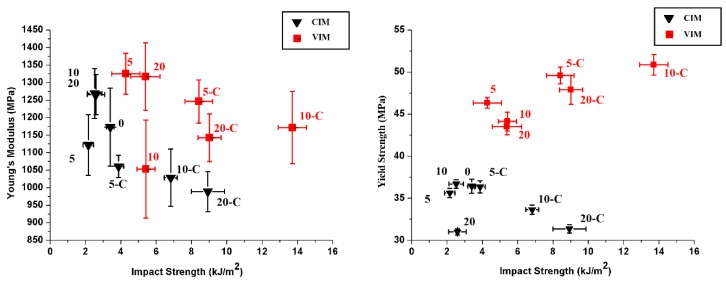
Impact strength VS Young’s modulus (**left**) and Impact strength VS yield strength (**right**).

The mechanical properties of the different samples are listed in Table 2 and Figure 2. As shown in Table 2 all mechanical properties of VIM-0 are much higher than others, because it consists of nearly all shish-kebab. Loading PET leads to the deterioration of shish-kebab due to the incompatibility between PP and PET. In the practice of recycling unavoidably blended polymers, CIM-0 and VIM-0 cannot be made; there is always a PET contamination. In order to give a clear comparison between the various blends, the VIM-0 is therefore not shown in Figure 2, which is used to select the blend with best mechanical performance by evaluating the balance of toughness and strength. It can be found that VIM-10-C possess highest impact strength and yield strength simultaneously, with an increment of 39% for yield strength and 440% for impact strength compared to CIM-10. Besides, Young’s modulus of VIM-10-C is not deteriorated and remains at the same level of CIM-0. Thus, we really found a new approach to optimize mechanical properties and maintain the balance of strength-toughness for immiscible PP/PET blend. 

Overall, it is obvious that samples processed by MFVIM (red square) possess better mechanical properties than CIM samples (black triangle), owning to the high mechanical performance of shish-kebab. Due to the elastic character of POE–*g*–MA, loading compatibilizer would be expected to lead to a decrease in modulus for both CIM and VIM samples. However, the introduction of shish-kebab via MFVIM can structurally compensate this loss of property to some degree. As for yield strength, a different trend is observed: Loading POE–*g*–MA has little influence on CIM samples but is beneficial for VIM samples. It is somewhat counterintuitive since the soft elastomer is supposed to decrease tensile strength [28]. One possible explanation for the increase of yield strength for VIM samples is that the reduced PET size, caused by compatibilizer, would promote the formation of shish-kebab instead of hindering it. As mentioned before, VIM-10-C possesses the highest impact strength of all samples. Unlike the monotonic increase of impact strength with increasing PET for CIM samples including POE–*g*–MA, it seems that an optimal content of PET, i.e., 10%, exists for VIM samples. To reveal the origin of this phenomenon, detailed characterization covering morphology and crystalline structure has been carried out for discussion.

### 3.2. Crystallization of PP Matrix

**Figure 3:**
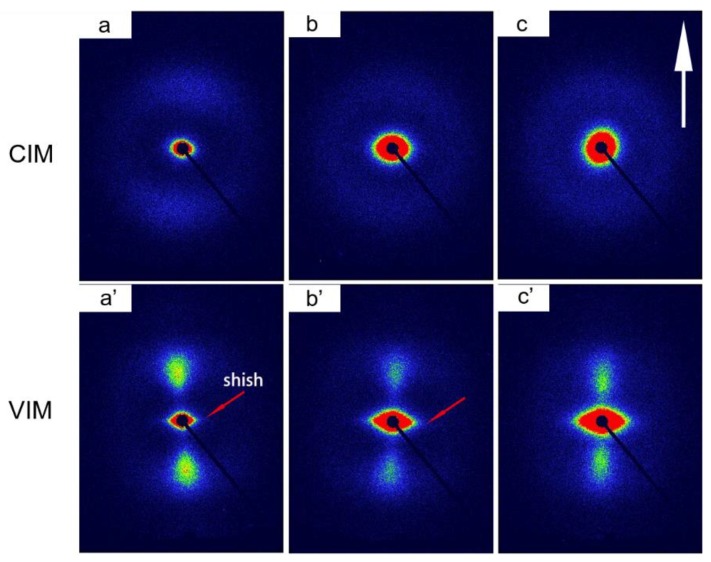
Typical 2D-SAXS patterns: (**a**,**a’**) is pure PP; (**b**,**b’**) is CIM/VIM-10; and (**c**,**c’**) is CIM/VIM-10-C. The white arrow depicts the flow direction.

MFVIM can fabricate samples with high content of shish-kebab for pure isotactic PP, which has been extensively studied in our previous work [20,26]. In the current work, 2D-SAXS study was performed to detect the existence of shish-kebab crystals after intense shear induced by MFVIM. Figure 3 depicts the characteristic 2D-SAXS patterns obtained for CIM and VIM samples. In general, all CIM samples show an isotropic halo in scattering intensity, indicating no obvious orientation of lamellar crystals, while for VIM samples, two vertical bulb-shaped lobs and two sharp triangular streaks (marked by red arrows) appear in meridional and equatorial directions, respectively. The vertical lobs in scattering intensity are characteristic for shish-kebab and correspond to the long period (L) of crystal kebabs. It is also depicted that the red circle in the center region of scattering intensity, indicating the existence of large-scale structure, becomes larger with the introduction of PET and POE–*g*–MA. For CIM/VIM-10-C, this large-scale structure, consisting of PET and POE–*g*–MA, is exactly the core-shell structure with PET encapsulated in POE–*g*–MA, which will be discussed later. Please note that the injection molded pure PP have typical “skin-core” structure with shear layer (or skin layer) consisting of shish-kebab and core layer consisting of spherulite. As shown in Figure S2 (Supplementary Materials), shear layer thickness of all VIM samples is much higher than that of the core layer, especially visible for pure PP. From this point of view, the existence of large content of shish-kebab in shear layer induced by MFVIM can also be confirmed.

**Figure 4:**
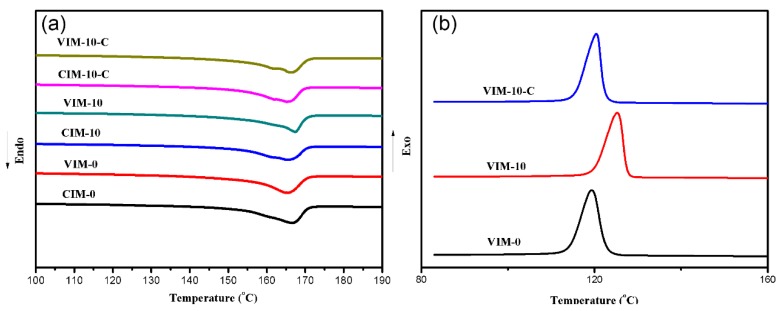
DSC curves: (**a**) heating process and (**b**) cooling process.

Thermal behavior of PP crystals was evaluated by DSC experiments. The heating and cooling curves are available in Figure 4 and related values are listed in Table 3. It is found that melting temperature (*T*_m_) for all samples is around 166 °C and shows no distinct difference. As for crystallization temperature (*T*_c_), the introduction of PET significantly increased it from 119.4 °C for pure PP to 125.3 °C for the sample containing 10% PET. However, after adding POE–*g*–MA, the *T*_c_ went back to the same level as pure PP at about 120 °C. The same results were also obtained in previous research [9,29] studying PP/PET microfibril composites. Authors attributed the increased crystallization temperature to the heterogeneous nucleation ability of PP on PET fibers, but this ability is inhibited by the presence of the compatibilizers that to some degree cover the PET fibers around the PP matrix. Combined with the current results we can rationally draw conclusions that PET will promote the crystallization of PP, regardless of its phase morphology (fibril or spherical), but only in the absence of compatibilizer. No matter how the crystallization initiates, the total crystallinity (*X*_c_) is not affected for all samples, as depicted in Table 3. 

**Figure 5:**
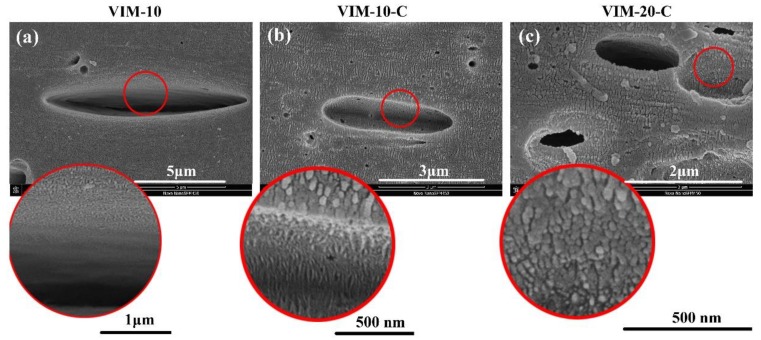
(**a**–**c**) Crystalline morphology of PP. Red circles are enlarged view of interface.

To obtain more insight into the crystallization behavior of the PP matrix, especially at the PP-PET interface, the interfacial morphology was observed by SEM, as depicted in Figure 5. Please note that all PET, POE–*g*–MA, and amorphous region of PP were etched by acid solution, leaving the holes and visible PP lamellar crystals on the matrix. Focusing on the interface of PP and PET (red circles and enlarged view), it is clear that for VIM-10, no lamellar crystals can be found and only a smooth area exists, indicating the poor adhesion between PP and PET without compatibilizer, which corresponds well with the limited impact strength. However, for VIM-10-C and VIM-20-C, lamellar crystals of PP matrix are discernible at the interface. That is to say, the adhesion of PP and PET was strengthened by interfacial crystallization of PP with the introducing of POE–*g*–MA. The proposed mechanism is that POE–*g*–MA would firstly cover the PET, then PP lamellar crystals could insert into POE–*g*–MA. After POE–*g*–MA was dissolved, the inserted lamellar crystals were finally visible, as shown in Figure 5b,c. Previous studies also showed a certain degree of miscibility of PP and POE. The effective anchoring of the PP crystallites into the POE phase can enhance the connection and facilitate the energy transfer between different phases [30,31,32]. 

### 3.3. Phase Morphology and Core-Shell Structure

**Figure 6:**
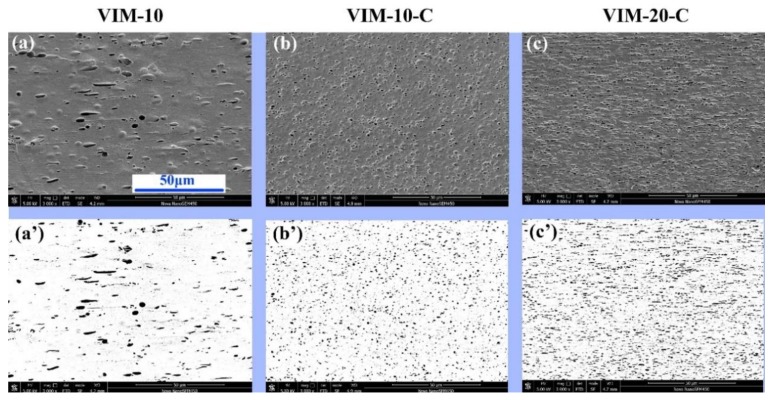
Phase morphology of the immiscible blend. First row is the original SEM photo while second row is the modified picture for the convenience of clear observation. Flow direction is horizontal.

It is widely recognized that a compatibilizer can significantly reduce the size of dispersed PET domains by reducing the interfacial tension due to the dual interaction of the compatibilizer on both PP and PET [8,14]. Corroborating results were obtained in this work as shown in Figure 6. In general, typical sea-island phase morphology of an immiscible blend with PET dispersed in a PP matrix was observed for all samples. Apparently, size of the PET domain (black dots in the second row of Figure 6) was reduced for VIM-10-C/VIM-20-C compared with VIM-10, and the dispersion became more uniform with the aid of POE–*g*–MA. In addition to the difference in dispersion density of PET domains for VIM-10-C and VIM-20-C, it is also observed that PET droplets are elongated along the flow direction for VIM-20-C while for VIM-10-C they remain spherical. This variation of phase morphology is induced by the high shear strength during MFVIM, as discussed later. 

**Figure 7:**
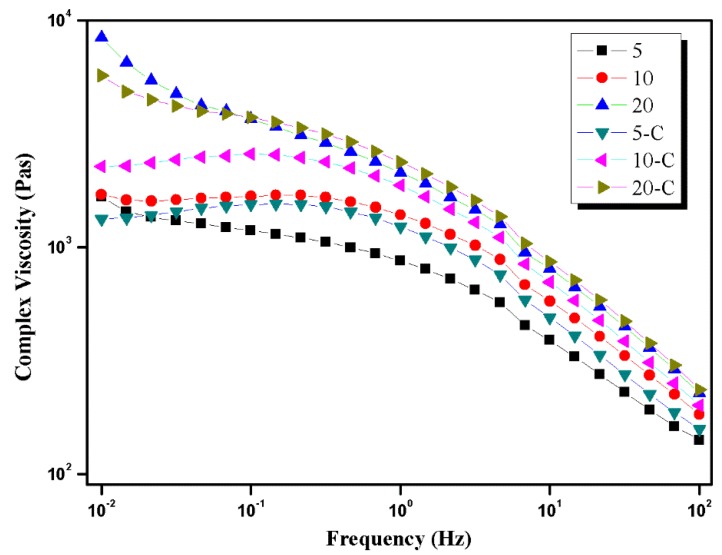
Complex viscosity of different blend.

Please note that the nozzle temperature during injection molding was set as 200 °C and PET was not fully melted at this temperature. Unmolten PET could eventually lead to the increase of viscosity with higher content of PET, as depicted in Figure 7. Meanwhile, loading POE–*g*–MA would also contribute to the higher viscosity due to its elastic character. These two reasons jointly contributed to the highest viscosity of the blend 20-C, generating the highest shear strength during MFVIM, which ultimately resulted in the formation of elongated PET domains. In addition, higher content can also give a chance to form a longer PET phase.

It was mentioned before that POE-g-MA would migrate to the interface of PP/PET to cover PET, then PP lamellae could insert into POE–*g*–MA, which has been indirectly proven in Figure 5. According to Hobbs et al. [33,34], the formation of the core-shell structure with one phase encapsulated by another can be predicted by a thermodynamic criterion concerning interfacial tension and spreading coefficient. For a ternary blend consisting of components 1, 2, and 3, the spreading coefficient $\lambda_{31}$ (component 3 as the shell and component 1as the core) can be calculated by:

(2) $$\;\lambda_{31}=\gamma_{12}-\gamma_{23}-\gamma_{31}\;$$

in which $\gamma_{12}$, $\gamma_{23}$ and $\gamma_{31}$ are the interfacial tension between each component pair (index 2 refer to matrix), calculated by:

(3) $$\;\gamma_{12}=\gamma_{1}+\gamma_{2}-4\left(\frac{\gamma_{1}^{d}\gamma_{2}^{d}}{\gamma_{1}^{d}+\gamma_{2}^{d}}+\frac{\gamma_{1}^{p}\gamma_{2}^{p}}{\gamma_{1}^{p}+\gamma_{2}^{p}}\right)\;$$

where $\gamma_{1}$, $\gamma_{2}$, $\gamma^{p}$ and $\gamma^{d}$ denote the γ values (surface tension) of each phase and their polar and dispersive components, respectively. Only if $\lambda_{31}$>0 can a core-shell structure with component 1 encapsulated by component 3 form. Kordjazi [13] observed the core-shell structure in PP/PET/SEBS–*g*–MA ternary blend, which corresponded well with the calculated result. 

In addition to the thermodynamic criterion, kinetic factors should also be considered. Li [35] reported that different blending methods (one-step or two-step) would influence the thickness of shell for PA/HDPE/EPDM–*g*–MA ternary blend due to the changed diffusion process of EPDM–*g*–MA. Likewise, it is difficult for all elastomeric compatibilizer (POE–*g*–MA) to diffuse in the melt to encapsulate PET during one-step blending, even though the thermodynamic criterion is satisfied. Thus, part of POE–*g*–MA will remain randomly dispersed in the PP matrix rather than covering the PET.

**Figure 8:**
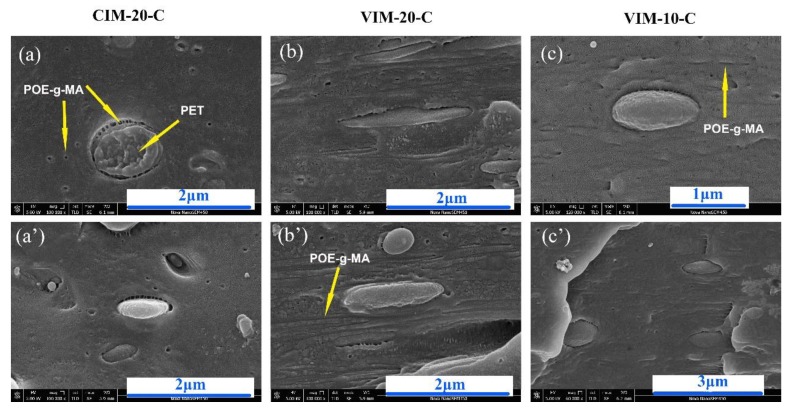
Core-shell structures. Arrows indicate the POE–*g*–MA removed by n-heptane. Flow direction is horizontal.

Above discussions were confirmed by the observation shown in Figure 8. A clear gap can be seen between PET and PP matrix in all pictures, which is caused by the removal of POE–*g*–MA by n-heptane. Based on this result, we can confirm the validation of the mentioned thermodynamic criterion and the existence of the typical core-shell structure. Besides, the shape of the core-shell structure corresponds well with the morphology observed in Figure 6. That is, the core-shell structure of VIM-20-C was elongated due to its high viscosity, while for CIM-20-C and VIM-10-C, it remains spherical. Outside the core-shell structure, some black dots and long grooves (marked by arrows) can also be observed. As discussed before, limited blending time inhibit the diffusion of all POE–*g*–MA to form a shell, resulting to the randomly distribution of POE–*g*–MA outside core-shell structure. High shear strength caused by MFVIM would also elongate POE–*g*–MA particles, causing the long grooves shown in Figure 8b. 

### 3.4. Toughening Mechanism

**Figure 9:**
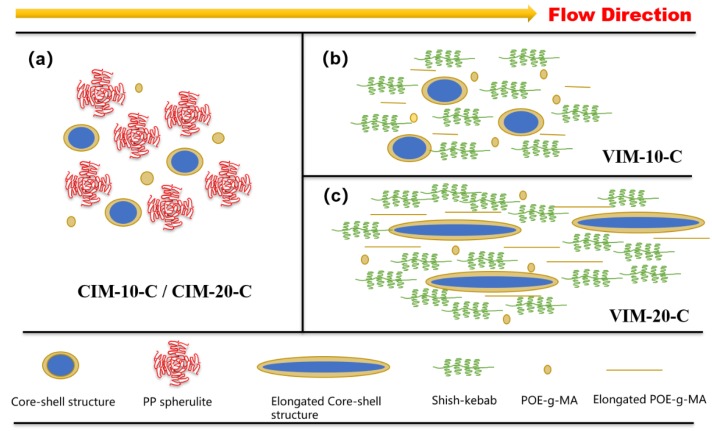
(**a**–**c**) Schematic drawing of phase morphology and crystalline structure for different samples. Note that the size of all symbols is not to scale.

For the sake of discussion, a schematic picture was drawn based on the above characterizations covering crystalline structure and phase morphology. As depicted in Figure 9a, a lot of PP spherulites are formed in CIM-10-C and CIM-20-C due to the relatively moderate shear force in conventional injection molding. This moderate processing condition is beneficial for the formation of the uniform spherical core-shell structure, and even the dispersed POE–*g*–MA outside core-shell structure is spherical. Thus, compared with CIM-10 and CIM-20, the impact strength for CIM-10-C and CIM-20-C improved to some degree (2.52 to 6.82 kJ/m^2^ and 2.59 to 8.93 kJ/m^2^, respectively). CIM-5-C also improved impact strength for CIM-5 from 2.16 to 3.87 kJ/m^2^. It is found that CIM-20-C possesses the highest impact strength, and this may be due to the fact that the diameter and quantity of core-shell structure is larger than that of CIM-5-C and CIM-10-C. 

As shown in Figure 9b,c, PP spherulites are converted into shish-kebab crystals due to the periodical high shear strength of MFVIM. High content of shish-kebab is supposed to dramatically increase the mechanical properties of pure PP, while for the immiscible blend, this enhancement is slightly limited due to the incompatibility. For both VIM-10-C and VIM-20-C, some of the randomly dispersed POE–*g*–MA are elongated into line-shaped structures with a high aspect ratio. Besides, the shape of most core-shell structures for VIM-20-C totally changed from spherical balls to elongated rods. This is caused by the high content of PET (20%) incorporated with POE–*g*–MA, resulting in the high viscosity value and more opportunity of touching and shearing for adjacent core-shell structures during injection molding. In general, the toughening effect of elongated elastomer particles oriented along flow direction is found to be not as well as spherical particles [36,37] due to the poor ability in initializing massive crazing and micro-voiding. Likewise, elongated core-shell structure with high aspect ratio is not beneficial for the fibrillation process [38] of core-shell particles when absorbing impact energy. Thus, compared with CIM-20-C, the impact strength of VIM-20-C shows nearly no improvement. On the one hand, shish-kebab can improve impact strength, while on the other hand, the elongated core-shell particles will weaken the toughening effect. The balance of these two effects jointly induced the unchanged impact strength of VIM-20-C, as compared with CIM-20-C, which is mainly toughened by uniform spherical core-shell particles. 

**Figure 10:**
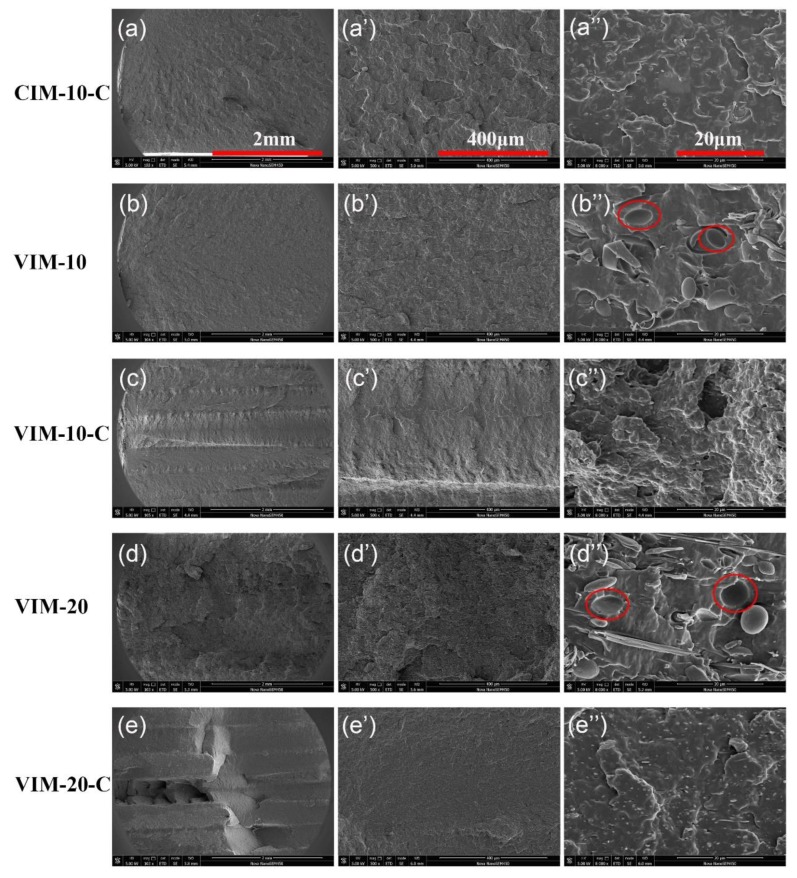
Impact fracture surfaces. (**a’**–**e’**) and (**a’’**–**e’’**) are magnified pictures. Impact direction is horizontal.

Impact fracture surfaces of five samples shown in Figure 10 were observed to obtain more insight into the toughening mechanism. Figure 10a,e are full views of the impact fracture surfaces. Clearly, a layered structure is found for VIM-10-C and VIM-20-C (Figure 10c,e), while the layering phenomenon is not visible in VIM-10 and VIM-20 (Figure 10b,d). The layers were formed during MFVIM process and each flow could induce one shear layer, which can be distinguished in PLM photos (Figure S2, Supplementary Materials). The different fracture mechanisms of shish-kebab in shear layer and spherulite in core layer caused the observed layering phenomenon. That is, shish-kebab with polymer chains highly oriented along flow direction can withstand high-speed tensile deformation, which facilitates the absorption of more impact energy and induces more plastic deformation than spherulites [39]. As for VIM-10 and VIM-20, this layering phenomenon is prohibited due to the poor interfacial adhesion. That is because the fracture process of VIM-10 and VIM-20 is mainly caused by interfacial debonding and crazing (see red circle in Figure 10b’’,d’’), which occurred prior to shish-kebab or spherulite absorbing plenty energy. 

CIM-10-C also lacks the layered structure (Figure 10a) because the shear layer of CIM samples is very thin compared with the core layer, thus the fracture process is dominated by the spherulites in the core layer. We can just see the “patches” resulting from crazing and micro-voiding in Figure 10a, but the fracture surface is not very coarse, suggesting that the absorbed impact energy is limited. However, the detailed fracture surface of VIM-10-C (Figure 10c’’) is rougher and coarser than other samples, which is in line with its highest impact strength. The uniform spherical core-shell particles can act as stress concentration point to induce local cavitation and crazing, which ultimately release triaxial tension and achieve shear yielding of matrix [38]. The joint action of core-shell particles and shish-kebab enables VIM-10-C the highest impact strength among all samples in this experiment.

The tensile process will also be briefly discussed for the sake of a full understanding. Generally speaking, core-shell structure has no distinct influence on tensile strength, but the reduced size of PET domains caused by compatibilization effect is beneficial to reduce the possibility of interfacial debonding during stretching process.

On the one hand, smaller size of PET domains caused by the formation of core-shell particles can reduce the risks of cavitation at PP/PET interface before shish-kebab reaches its highest stress, causing a higher yield strength. On the other hand, higher content of randomly dispersed isolated POE–*g*–MA particles can also induce voids during stretching, causing a decrease in modulus and strength [40]. The competition of these two effects may explain why VIM-10-C possesses the highest yield strength. 

## 4. Conclusions

The current research aims at providing fundamental insights for reprocessing immiscible PP/PET blends from inseparable PP and PET components. The objective is to improve the mechanical performance so that the application value of PP/PET blends in the mechanical recycling process increased. We found that the compatibilization effect of POE–*g*–MA on PP/PET blends consisted in the formation of a core-shell structure by diffusion of the POE–*g*–MA into the PP matrix to encapsulate the PET owning to the thermodynamic criterion. The uniform spherical core-shell particles can reduce the size of PET domains and therefore improve impact strength due to its toughening effect. Besides, we used a novel MFVIM to convert PP spherulites into highly oriented shish-kebab crystals, which have been demonstrated to be effective in improving mechanical properties. However, high shear strength of MFVIM causes the formation of many elongated core-shell particles in samples containing higher content of PET (20%), and this elongated core-shell particle has little improvement on impact strength when compared with a spherical one. Finally, we found that the joint action of spherical core-shell particles and shish-kebab crystals enabled sample VIM-10-C the best performance with a balance between strength and toughness, of which the yield strength and impact strength improved 39% and 440%, as compared to CIM-10. In short, we demonstrated a new approach to optimize the mechanical properties of the immiscible PP/PET blend via incorporation of shish-kebab and core-shell structure. This work is of practical significance especially in recycling process of PP/PET solid wastes.

## Figures and Tables

**Table 1:** Composition of the blends.

Material	PP/PET wt %	POE–*g*–MA wt %
Pure PP	100/0	0
5	95/5	0
5-C	93.5/4.9	2
10	90/10	0
10-C	86.4/9.6	4
20	80/20	0
20-C	75.2/18.8	6

**Table 2:** Mechanical properties.

Samples	0		5		5-C		10		10-C		20		20-C	
	CIM	VIM	CIM	VIM	CIM	VIM	CIM	VIM	CIM	VIM	CIM	VIM	CIM	VIM
Yield strength/MPa	36.42 ± 0.84	58.46 ± 0.87	35.61 ± 0.56	46.33 ± 0.64	36.35 ± 0.73	49.6 ± 1.00	36.69 ± 0.51	44.12 ± 1.11	33.63 ± 0.55	50.87 ± 1.23	30.99 ± 0.37	43.51 ± 0.99	31.34 ± 0.48	47.89 ± 1.70
Young’s modulus/MPa	1173 ± 112	1421 ± 120	1122 ± 87	1326 ± 58	1061 ± 32	1247 ± 62	1269 ± 71	1181 ± 96	1029 ± 82	1172 ± 103	1265 ± 58	1318 ± 96	989 ± 57	1143 ± 68
Impact strength/kJ.m^-2^	3.40 ± 0.22	18.20 ± 1.00	2.16 ± 0.29	4.28 ± 0.79	3.87 ± 0.3	8.41 ± 0.78	2.52 ± 0.41	5.43 ± 0.51	6.82 ± 0.37	13.71 ± 0.80	2.59 ± 0.50	5.4 ± 0.83	8.93 ± 0.94	9.02 ± 0.65

**Table 3:** Thermal properties during heating and cooling.

Samples	Thermal properties			
	*T*_m_/°C	*T*_c_/°C	ΔH/J·g^−1^	*X*_c_/%
CIM-0	166.70	-	76.39	36.5
VIM-0	165.66	119.36	75.83	36.3
CIM-10	165.57	-	70.02	37.2
VIM-10	167.38	125.25	73.05	38.8
CIM-10-C	165.32	-	66.32	36.7
VIM-10-C	166.43	120.40	68.95	38.2

## Supplementary Materials

The following are available online at http://www.mdpi.com/2073-4360/10/10/1094/s1. Figure S1: Mechanical properties of samples with various content of POE-g-MA, Figure S2: PLM photos of different samples.
