# Supplementary material for: New Approach to Optimize Mechanical Properties of the Immiscible Polypropylene/Poly (Ethylene Terephthalate) Blend: Effect of Shish-Kebab and Core-Shell Structure
Source: Polymers (Basel). 2018 Oct 2;10(10):1094. doi: 10.3390/polym10101094 (PMC6403558; doi:10.3390/polym10101094)
Supplement: Supplementary file 1 [file polymers-10-01094-s001.pdf]

polymers

Supplementary Materials

New approach to optimize mechanical properties of the immiscible Polypropylene / Poly (ethylene terephthalate) blend: Effect of shish-kebab and core-shell structure

Yingxiong Wang [1], Dashan Mi [1,2], Laurens Delva [2], Ludwig Cardon [2], Jie Zhang [1,*], Kim Ragaert [2,*]

1. College of Polymer Science and Engineering, State Key Laboratory of Polymer Materials Engineering Sichuan University, Chengdu, China

2. Centre for Polymer and Material Technologies, Department of Materials, Textiles and Chemical Engineering, Faculty of Engineering and Architecture, Ghent University, Technologiepark 915, Zwijnaarde 9052, Belgium

Correspondence: zhangjie@scu.edu.cn (J.Z.); kim.ragaert@ugent.be (K.R.)

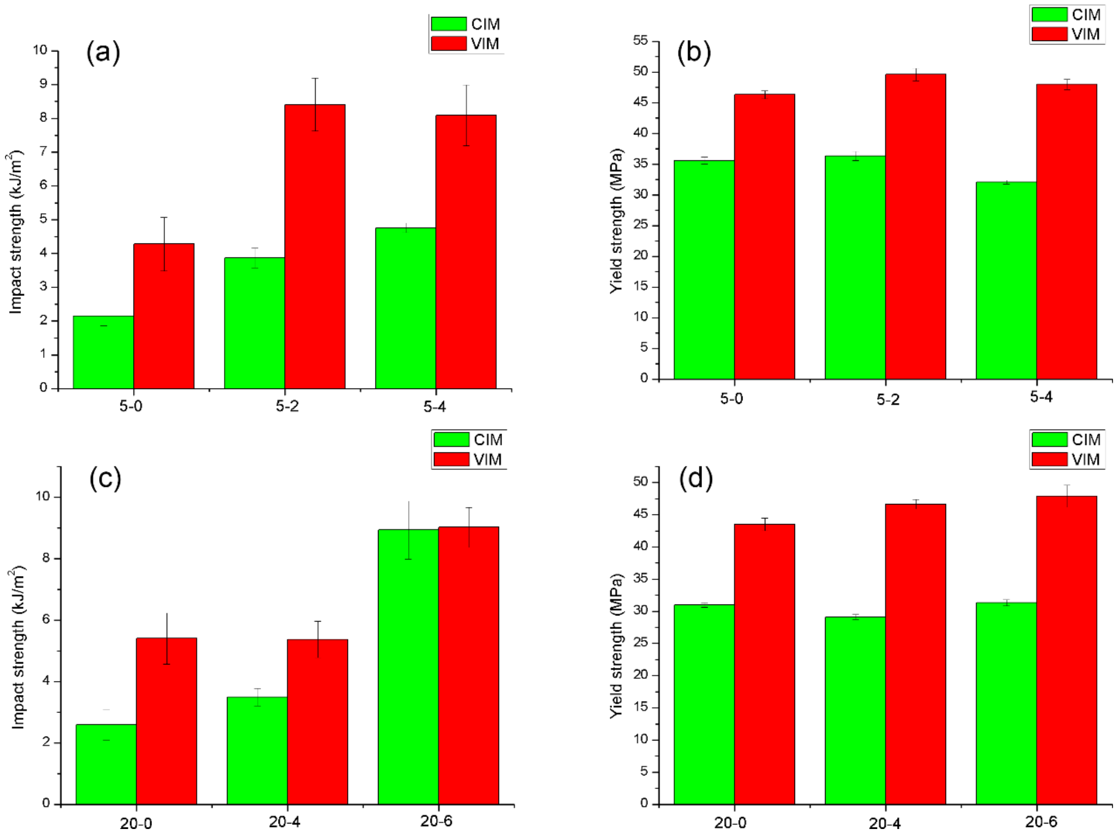

Figure S1 Mechanical properties of samples with various content of POE-g-MA. 5-2 means sample containing 5% PET and 2% POE-g-MA and rest are same.

As shown in Figure S1, impact strength for sample CIM/VIM-5-2 and 5-4 has no distinct differences, but the values are much higher than that of sample CIM/VIM-5. That is to say, the extra 2% POE-g-MA for samples containing 5% PET has no obviously improving on impact strength, especially for VIM sample. Thus, loading only 2% POE-g-MA for samples

containing 5% PET is enough. However, compared with CIM/VIM-20, only adding 6% POE-g-MA can induce obviously toughening effect but VIM-20-4 shares nearly same value with VIM-20. It is necessary to load 6% POE-g-MA instead of 4% for samples containing 20%. The chosen value of content for POE-g-MA is rational in this experiment. We may speculate that for samples containing 20% PET, 4% POE-g-MA is not much enough to encapsulate PET to form uniform core-shell structure while higher content of POE-g-MA (6%) can realize this goal. Analogously, 2% POE-g-MA is much enough for sample CIM/VIM-5-C to form core-shell structure, not even to say loading 4% POE-g-MA.

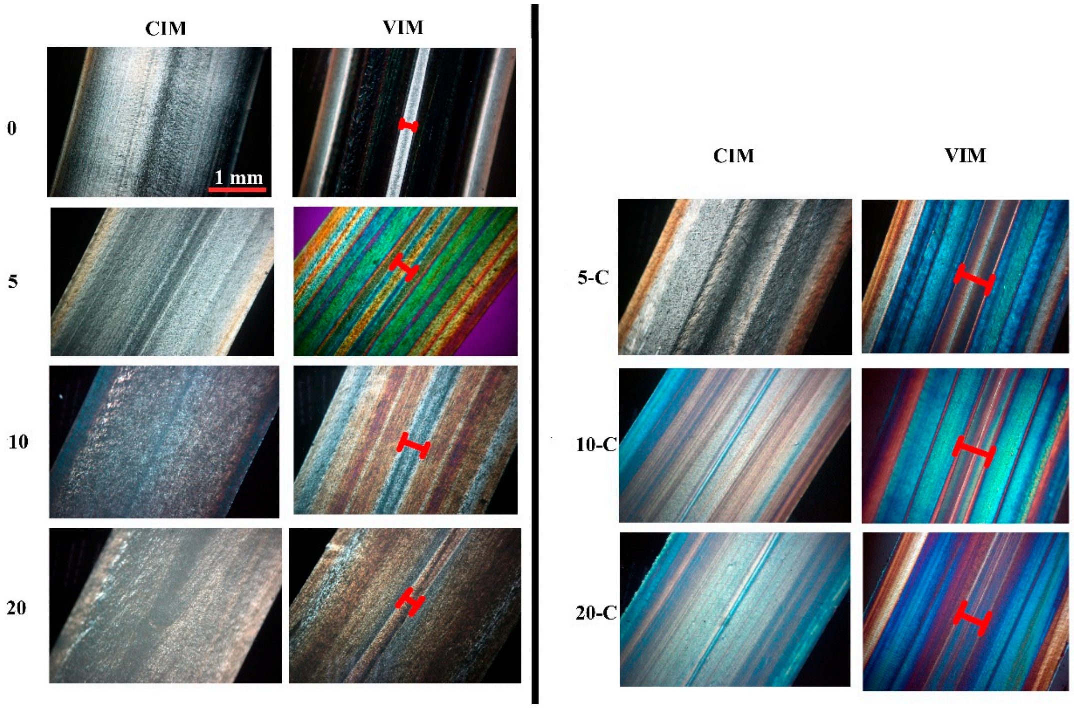

Figure S2 PLM photos of different samples. Core layers are marked by red lines.

Typical "skin-core" structure of injection molded part is discernable for all samples in Figure S2. It is supposed that shear layer is consisted of shish-kebab while core layer is consisted of spherulite for PP matrix. Clearly, shear layer nearly occupies whole range of VIM-0, which resulted to the high mechanical properties of VIM-0. Though loading PET makes this "skin-core" structure a little bit difficult to distinguish, it is also clear that core layer thickness significantly decreased, as marked by red arrow. Based on this, we can conclude that samples with high content of shish-kebab that exists in shear layer, has been successfully fabricated by MFVIM.
